# Effect of short message service delivered health education messages on parental barriers to childhood immunisation in Borno State, Nigeria: a quasi-experimental study

**DOI:** 10.4314/ahs.v25i4.7

**Published:** 2025-12

**Authors:** Dathini Hamina, Siti Khuzaimah Ahmad Sharoni, Robert Kever, Joseph-Shehu Elizabeth

**Affiliations:** 1 Centre for Nursing Studies, Faculty of Health Sciences, Universiti Teknologi MARA, UiTM Selangor, Puncak Alam Campus, Selangor 42300, Malaysia; 2 Department of Nursing Science, Faculty of Allied Health, University of Maiduguri, Maiduguri 600104, Nigeria; 3 Department of Nursing Science, National Open University of Nigeria, Abuja, Nigeria

**Keywords:** Vaccines, Immunisation, Barriers, Nigeria

## Abstract

**Background:**

In Nigeria, barriers to immunisation such as myths and misconceptions arising from belief, lack of knowledge and social or family influence exist. The aim of this study was to determine the effect of Short Message Service (SMS) delivered health education messages on barriers to childhood immunization in Maiduguri, Borno State, Nigeria.

**Methods:**

The study is a pre-and-post-test quasi-experimental study conducted among 262 parents of infants in Maiduguri, Nigeria. One SMS-delivered health education message was sent to parents of infants one week to the immunisation date for the 6th, 10th, and 14th-week immunization schedule. The immunisation fact messages were adapted from UNICEF and translated to Hausa language. At baseline and endline, the Searching for Hardship to Shots (SHOT) survey questionnaire was administered to determine barriers to immunization. Data collected were analysed using Wilcoxon signed rank test. The results were significant if the p-value was <0.05 at a 95% confidence interval.

**Results:**

Findings of the study indicate that overall, SMS delivered health education significantly (p-value = 0.001) reduced total barrier to parental immunization. Additionally, interventions significantly reduce access and importance (p-value = 0.001) barriers, although barriers regarding parental concern was reduced, it was however, not statistically significant (p-value = 0.055).

**Conclusion:**

The study affirms the important role of mobile communication health technology in improving outcome of immunization in Nigeria.

## Introduction

Infectious diseases are the leading cause of morbidity and mortality among infants, especially after the first six weeks of life^1^. According to evidence, these infectious diseases are of public health concern with adverse consequences on world economies and increasingly threatening health security for many nations^2^,^3^. Infectious diseases account for about 5.3 million infants and child mortality globally^4^. Research indicates that 99% of such deaths occur in developing countries largely because of poor immunisation coverage^4^. Accordingly, evidence opined that vaccines effectively prevent approximately 40% of global mortality of which 52% of these mortalities happen in Africa^5^,^6^. The high proportion of Vaccine Preventable Diseases (VPD) reported in African countries is concerning and calls for concerted effort to ensure that vaccines are available and administered to children to protect their health and prevent mortality^5^. Immunization has been recognized as a cost-effective strategy for protecting the health and well-being^2^,^3^. According to the World Health Organisation, for every single dollar spent on immunization, there is a saving of 44 dollars that would have otherwise been used for managing infectious diseases^7^.

Despite the known benefits of immunisations, several factors impede parents or caregivers from accessing immunisation services for their children^8^. According to evidence, barriers to immunisation is multifaceted in context and differs over time and place, influenced by factors such as satisfaction, trust, and convenience in accessing it^9^-^11^. There have been calls by different researchers on the need to develop interventions that would reduce barriers to immunisation among children for improved coverage and reduction of VPDs^12^-^14^. In Nigeria, different barriers to immunisation such as myths and misconceptions arising from belief, lack of knowledge and social or family Influence exist^8^,^11^,^13^. These factors have made optimal immunisation coverage in Nigeria for children to be a mirage. It is in this regards that this study was designed to determine the effects of SMS-delivered health education messages on barriers to infant immunisation in Borno state, Nigeria.

## Methods

### Study design

It was a pre-and-post-test quasi-experimental study.

### Study population

The population for this study were parents of infants who are four weeks or less in Maiduguri, Borno state, Nigeria.

### Eligibility criteria

#### Inclusion criteria

Participants were recruited into the study if they were:

1. Parents of infant four weeks or less.
2. Parents or caregiver with an active mobile phone.
3. Parents indicates no intention to relocate within six months.
4. For mothers with twin babies, the index child was the youngest.

#### Exclusion criteria

The following was used to exclude participants from the study.

1. Participant who are not of legal age of 18 for consenting.
2. Participants who cannot read messages in English or Hausa or at least get someone within the household who can read and interpret the message.

### Research setting

The study location for this study was Maiduguri, Borno state, Nigeria. It is situated at latitudes 100N and 140N and longitude 11030′E and 14045′E, established in 1976. Borno state is divided into 27 units called the Local Government Area (LGA) council. For this study, Maiduguri Metropolitan Council (MMC) was used. The study location has an estimated population of 5.2 million of which 758,000 resides in Maiduguri metropolitan council^15^. There are an estimated 2.7 million active mobile phones, most of which are in Maiduguri, which made it viable for the intervention to be conducted.

### Intervention

The intervention for this study was SMS-delivered health education messages adopted from UNICEF immunisation fact messages^16^ (UNICEF, 2019). The SMS-delivered health education messages were sent to parents of infants in their preferred language (Hausa or English) one week to the immunisation due date three immunisation schedules. At baseline and endline, the SHOTS questionnaire (accessed at: http://www.shotsurvey.org/) was administered to assess barriers to immunisation. The SHOTS questionnaire comprises of 23 items divided into three subscales, the access, concerns, and importance subscales. The instrument has an overall Cronbach's Alpha reliability coefficient of 0.93 with the reliability coefficient of the subscales as 0.92, 0.81, and 0.75 for the access concern and importance subscales respectively (http://www.shotsurvey.org/). The SHOTS questionnaire was used in this study because its constructs address the major barriers identified for immunization in the study setting^11^,^13^.

### Outcome measure

The outcome measure for this study was parental barriers to childhood immunisation.

### Calculation of sample size

Sakpal et al.^17^ formula for sample size calculation and the proportion from the study of Ibraheem et al.^14^ was used. n = [(Zα/2 + Zβ)2 × {(p1 (1-p1) + (p2 (1-p2))}]/(p1-p2)2.

Additionally, an attrition rate of 20% was used. This resulted in 262 participants for the study.

### Randomisation

A simple random sampling technique was used to randomly allocate the intervention. There was no randomisation at the level of participant enrolment due to the nature of the intervention. Hence participants were purposively recruited based on eligibility criteria.

### Sampling method and allocation concealment

A simple random sampling technique was used alongside opaque envelopes to conceal the allocation in the selection of healthcare facilities for this study. To achieve this, all healthcare facilities were listed each on a separate paper and inserted into sealed opaque envelopes and shuffled thoroughly. A volunteer picked one envelope to determine the facility that was included for this study. On the other hand, purposive sampling was used to enroll participants into the study. A total of 262 respondents regardless of gender were enrolled into the study.

### Blinding

Both the healthcare workers and statistician were blinded for this study. Hence, it was a double blinded study.

### Statistical analysis

Data were analysed using the Statistical Package for the Social Sciences version 28 (IBM SPSS, 2022, USA). For the socio-demographic data, frequencies, percentages, means and standard deviations were used. Because of the outcome of the normality test, Wilcoxon signed rank was used to determine the effects of the intervention on barriers to immunisation. The results were significant if the P-value was <0.05 at 95% confidence interval.

## Ethical approval

Ethical approval was obtained from Borno State Health Research and Ethics Committee (129/22) and Universiti Teknologi MARA Research and Ethics Committee REC/07/2023(PG/MR/232).

## Results

### Response Rate

A total of 262 participant that meets the eligibility criteria and were recruited to participate in this study. This represents 100% response rate for this study.

### Participants enrolment and recruitment

Recruitment of participant for this study began on the 28th of July 2023 and ended 13th December 2023. A total number of 92 respondents completed the study. This because despite the promt through SMS the parents failed to return. This further buttress the challenge with coverage of immunisation in Nigeria. A detailed flow chart of the respondents is as shown in figure I

**Figure 1:**
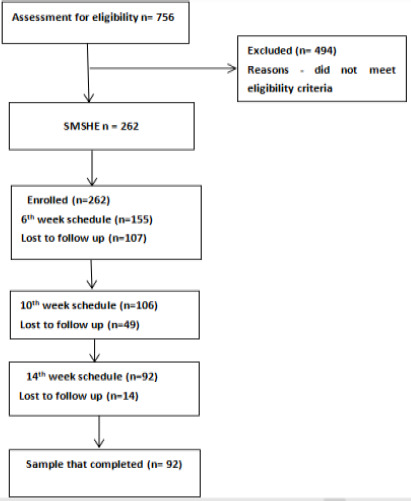
flow chart

### Socio-demographic characteristics

Table 1 reveals that mean age of respondents was 29± 7. Ninety-five percent of the research participants were of Islamic faith. With regards to educational attainment, majority (36.3%) had secondary school qualification while those with university education were the least representing 5.3%. Additionally, the reported mean income of the respondents was 284,047 naira (176.7 USD). Lastly, most (62.6%) of the deliveries occur in government owned facilities, only 3.8% happened at home the rest 33.6% were in privately owned health facilities.

**Table 1:** Socio-demographic data (N= 262)

Variable	Frequency (n)	Percentage (%)
**Age**		
Mean (SD)	29±7	
**Religion**		
Muslims	249	95
Christians	13	5
**Education level**		
No formal education	93	35.5
Primary	25	9.5
Secondary	95	36.3
Post Secondary	34	13
University	14	5.3
**Income**		
Mean	N284,047*(176.7 USD)	
**Place of delivery**		
Government facility	164	62.6
Private facility	88	33.6
Home	10	3.8

* N1607.5 = 1USD (12/8/2024)

Result on Table 2 shows the effect of SMS-delivered health education program on parental access to childhood immunisation. Result indicates that compared to the pre and post-test, the mean positive ranks was 49.75 and the mean negative ranks of 31.22 this indicates that intervention improved parental access to immunisation. This improvement was found to be statistically significant with a p value of 0.001. This therefore indicates that SMS-delivered health education significantly improved parental access to immunisation.

**Table 2:** Effect of SMS-delivered health education on parental access to childhood immunisation

		Mean Rank	Sum of Ranks	Z	Asymp. Sig. (2-tailed)
	Negative Ranks	31.22^a^	905.50		
access2 – access				-4.158^b^	0.001
	Positive Ranks	49.75^b^	2835.50		

Wilcoxon Signed Ranks Test

Results of the SMS-delivered health education intervention reveals when the baseline and endline results were compared, there was a positive mean rank score of 48.08 compared to the mean negative rank of 33.88. Although this indicate the positive effect of the intervention in reducing parental concerns, it was however, not statistically significant (p-value 0.055).

With regards to how parental understanding of the importance of immunisation affect acceptance of childhood immunisation. Results as shown on Table 4 indicates a positive mean rank score of 39.49 compared to the negative mean rank score of 21.52. this indicates that SMS-delivered health education improved parental knowledge on the importance of immunisation. This improvement was found to be statistically significant with a p-value of 0.001.

**Table 4:** Effect of SMS-health education on parental understanding of the importance of childhood immunisation

		Mean Rank	Sum of Ranks	Z	Asymp Sig. (2-tailed)
importance2 – importance	Negative Ranks	21.52	473.50	-4.058	0.001
	Positive Ranks	39.49	1737.50		

Wilcoxon Signed Ranks Test

The overall effect of the SMS-delivered health education messages on parental access, concern and importance barriers to immunisation reveals the overall positive mean ranks of 59.02 compared to the overall negative mean ranks of 26.18. This indicates that intervention reduced barriers parents face in getting their infants immunised. This overall improvement was found to be statistically significant with a p-value of 0.001 as shown on Table 5.

**Table 5:** Effect of SMS-health education on parental total barrier childhood immunisation

		Mean Rank	Sum of Ranks	Z	Asymp. Sig. (2-tailed)
ts2 – ts	Negative Ranks	26.18	995.00	-4.123	0.001
	Positive Ranks	59.02	3010.00		

Wilcoxon Signed Ranks Test

## Discussion

Adequate immunisation coverage is a cost-effective strategy for the prevention of infant and childhood morbidity and mortality^18^. This according to Adesina et al.^19^ is a consequence of low immunity to diseases that often-characterized children within this age bracket. This underpins the importance of ensuring that all children are adequately immunised. To the researchers' knowledge, this study provides the first set of evidence that determine the effect of SMS-delivered health education messages on barriers to childhood immunisation in Nigeria.

Finding from the study reveals the mean age of 29 ± 7 years which indicates that majority of participant are within the age of 22-36 years with the mean annual income of N284,047 (176.7 USD). The presence of complex emergencies and its attendant effect on the livelihood of people in Borno state, Nigeria might be the reason for the exceptionally low annual income recorded in this study. According to evidence, most of the inhabitants of Borno state are Agrarians and businessmen who often engage in cross border trade with neighbouring country (Niger and Chad Republic). The insecurity has made access to farms impossible with substantial proportion of the population leaving their ancestral lands to the state capital for security and the inability to engage in trade.

With regards to the ethnic group and religious affiliation of the participants, majority of the respondents were reported to be of Kanuri extraction and practice Islam. According to Harvard Divinity School, Islam came to Nigeria as early as the nineth century majorly through trade and migration through the regions of Kanem Borno^20^. It was therefore not surprising to observe that a greater proportion of the respondents for this study were Muslims. With regards to the place of delivery, result indicates that majority of women gave birth at government owned facilities. This could be due to the free maternal and child health services (including delivery) policy of Borno state Government and indeed many states in Nigeria^21^,^22^.

Results of this study on the effect of SMS-delivered health education message on barriers to immunisation reveals that, intervention led to a statistically significant finding in reducing barriers parents facing in getting their infants immunized in Maiduguri, Borno state, Nigeria. This finding is in line with the study of Babarinde and Atulomah^23^ who found that health education program was instrumental in reducing barriers to immunisation. According to Sato^12^, there is a widespread lack of knowledge of immunisation among parents. This position was also affirmed by Ignis and Tomini^10^ who reported in their study that lack of knowledge on immunisation is mostly cantered around vaccine safety, importance of vaccines and adverse event following immunisation. An intertwine of these factors eventually develops into vaccine misconception or helps to sustain already prevalent misconception on immunization in the community. It was interesting to see how this intervention was instrumental in reducing these barriers. Although overall barrier to immunisation was reduced, it was however, found to be statistically not significant in reducing parental concern. It could be an indication of the need for a more sustained approach as change in behaviour requires consistency^13^,^24^. Additionally, as the finding of this study indicates, majority of the participant had low level of education, and the assertion by evidence that lack of knowledge is an impediment to immunisation^12^. This could be part of the factors that contributed to this finding. On the other hand, the intervention significantly reduces parental access barrier to childhood immunisation. The improvement in parental access to vaccines could be attributed to the health education messages that serves as a reminder to parents.

According to a research study, forgetfulness is one of the major impediment parent encounters to their children immunisation^25^.

Additionally, the statistically significant result obtained with regards to the lack of knowledge of the importance of vaccines is a further affirmation of the lack of knowledge on childhood immunisation in this aspect. According to evidence, lack of knowledge of the usefulness of childhood vaccines among parents and caregivers of children is a major impediment to immunization in Nigeria^26^. The health education message intervention was designed to provide immunization fact messages to parents of infants. Part of the information provided revolves around understanding the importance of vaccines in preventing infectious diseases and mortality. Additionally, the information were delivered in a convenient manner through mobile phones. According to evidence, mobile health technologies are effective tools for the dissemination of safe and timely information for health preventive actions^27^. This is because recipients can at any time refer to the information to make sense of it or seek clarification from a trusted health provider of their choice. Additionally, the translation of the messages to Hausa language could be a factor for that resulted in the overall success of the intervention. According to evidence, translation of research instrument and health education messages to common language enhance comprehension and therefore improves healthcare service utilization^28^,^29^.

## Limitations of the study

Barriers to immunisation assessed were parent- reported barrier to immunisation. Additionally, the study was limited to the 6th, 10th, and 14th week immunisation schedule, small sample size, and high drop-out rate, the generalisation of the study findings to the entire schedule and population needs to be further evaluated.

## Summary and conclusion

The study was designed to evaluate the effect of SMS-delivered health education message on parental barriers to childhood immunisation in Nigeria. A one-group before-and-after quasi-experimental designed using 262 respondents was used. One SMS health education message was sent to parents of infants at one week to the immunisation due date for the 6th, 10th and 14th week immunisation schedule. At the end of the intervention, SMS delivered health education messages was found to have significantly reduce access and lack of knowledge of the importance of immunisation barriers parents encounter in the process of getting their infants immunised. On the other hand, although intervention reduced barriers associated with concerns, it was however, not statistically significant. Overall, however, SMS delivered health education statistically reduced parental barriers to immunisation in Borno state, Nigeria. The study therefore concludes that actively engaging parents of infants with immunisation fact messages can significantly reduce barriers to immunisation.

## Implication

The use of SMS delivered health education messages through mobile phones has demonstrated that it is an excellent medium for the dissemination of health education to parents especially in location that have vast population of active mobile users.

## Figures and Tables

**Table 3:** Effect of SMS-health education on parental concern to childhood immunisation

		Mean Rank	Sum of Ranks	Z	Asymp. Sig. (2-tailed)
concern2 – concern	Negative Ranks	33.88	1287.50	-1.917	0.055
	Positive Ranks	48.08	2115.50		

Wilcoxon Signed Ranks Test

## Data Availability

Dataset used for this study can be obtained from the corresponding author upon request for ethical use.
